# Morphological diversity of saber‐tooth upper canines and its functional implications

**DOI:** 10.1002/ar.25458

**Published:** 2024-04-22

**Authors:** Caitlin D. Shelbourne, Stephan Lautenschlager

**Affiliations:** ^1^ School of Geography, Earth and Environmental Sciences University of Birmingham Birmingham UK; ^2^ Lapworth Museum of Geology Birmingham UK

**Keywords:** canine teeth, functional morphology, geometric morphometrics, saber‐tooth, Smilodon

## Abstract

Elongated upper canine teeth, commonly known as saber‐teeth, have evolved three times within the sub‐order Feliformia. The species that wielded them flourished throughout the Cenozoic and have historically been separated into two morphological groups: the dirk‐tooths with longer, flatter canines, and the scimitar‐tooths with shorter, serrated teeth. However, quantitative morphological analysis has not been conducted on these teeth to determine the true amount of diversity within the group, and how the upper canine morphology of extant feliforms compared to their extinct relatives has also not been explored. Using Geometric Morphometric analysis, it is shown that saber‐tooth upper canine morphology is exceptionally diverse, with no extant clade having all its members occupy the same morphospace based on tooth length and curvature. Instead, a neutral basal morphospace is observed for all groups and diversification from this basal position is seen as species become more derived. A distinct and consistent scimitar tooth morphology is also not observed within the morphospace. When compared with extant taxa, several saber‐tooth species are seen to be morphologically similar to extant feliforms, several of which exhibit novel dietary strategies in comparison to the obligate carnivore felids. Biomechanical analyses of different actual and theoretical tooth shapes demonstrate that saber‐teeth upper canines further represent a functional compromise between sharpness, curvature, and length on the one hand, and robustness and material investment on the other.

## 1 INTRODUCTION

The long upper canine teeth of Cenozoic saber‐toothed predators are widely recognized as impressive weapons in both the scientific and wider communities, cementing the species that possess them as the likely apex predators of their respective ecosystems. However, despite their apparent ubiquity, the morphology of these teeth is diverse across the clades that have evolved them, as is the taxonomic position of the species they belong to. Elongated upper canine teeth have evolved four separate times in mammalian clades (Emerson & Radinsky, 1980), and in five clades within the Carnivora suborder Feliformia (Christiansen, 2012). Kurtén (1968) was the first to categorize the morphology of the saber‐tooth upper canine and did so into two general groups; the “dirk‐tooths” which have very long, moderately laterally flattened teeth with fine or no serrations, and the “scimitar‐tooths” whose teeth are shorter, more laterally flattened and with coarse serrations. Traditionally, each of the two classifications were restricted to a singular tribe of the subfamily Machairodontinae, with the species of the tribe Smilodontini being considered “dirk‐toothed”, and the species of the tribe Homotherini considered “scimitar toothed”. However, these classifications evolved to become broad taxon‐independent ecomorphs—groups of morphological characteristics that define an ecological niche (Antón, 2013). The expansion of these definitions allowed for the tooth morphologies to become associated with particular non‐dental characters, with the scimitar‐toothed morphology being more gracile and designed for speed with long limbs and a digitigrade posture (like that of a modern lion), and the dirk‐toothed morphology being opposite, with more robust skeletons, shorter limbs and plantigrade or semi plantigrade feet (like a modern bear) overall making a muscular animal designed for strength over speed (Martin, 1980).

Within feliforms, elongated upper canine teeth are found across five clades, and despite the popularity of the term “saber‐tooth cat”, only three of these clades actually fall within the family of “true cats”, Felidae. The family Nimravidae (Cope, 1880) is where saber‐toothed feliforms appear first, with the species *Dinictis felina* (Leidy, 1854) originating in the late Eocene, approximately 35 Mya (Bryant, 1996). Members of this clade are found throughout North America, Europe, and Asia (Bryant, 1996). They generally follow a “cat‐like” body plan, dentition, and cranial morphology, but notably, not all members possess the double‐chambered, ossified, auditory bulla that is diagnostic of Feliformia (Turner & Antón, 1997). This discrepancy has earned the group the moniker of the “false saber‐toothed cats” and has led some to place Nimravidae within Caniformia (Flynn & Galiano, 1982), though the overall consensus remains that the group should be considered part of Feliformia. The last members of Nimravidae died out at the end of the Oligocene, approximately 23 Mya (Bryant, 1996), meaning that they are the only clade included that did not overlap temporally with other “saber‐tooths”.

Perhaps some of the most extreme saber‐teeth seen in the feliforms exist within the family Barbourofelidae (Morlo et al., 2004), a sister taxon to Felidae that is considered to be the late‐surviving lineage of Nimravidae by some (Barrett, 2021). The earliest members of this clade originate in the late Oligocene (Piras et al., 2013), but they rise to prominence in the mid‐Miocene (approximately 16 Mya) across Africa, Eurasia, and North America (Robles et al., 2013). The barbourofelids display the most derived cranial features associated with dirk‐toothed species, with strongly exaggerated bone flanges on their mandibles and other cranial adaptations to maximize jaw gape (Antón, 2013). The barbourofelids die out approximately 7 Mya, and throughout their temporal range, they coexisted with species from all three clades within the “true” saber‐toothed cats of the clade Machairodontinae.

The felids of the clade Machairodontinae include species commonly referred to as saber‐toothed cats. They are separated into three main clades/tribes, Homotherini, Metailurini and Smilodontini. All machairodontins have their origins in the Middle Miocene with *Pseudaelurus quadridentatus* considered to be the first representative of the clade (Salesa et al., 2012). The tribe Homotherini diverges from this lineage first, at approximately 13 Mya (Piras et al., 2013) and homotherines persisted across Europe, North and South America and Africa until the Late Pleistocene (Reumer et al., 2003).

The metailurines break the mold when it comes to saber‐tooth morphology, maintaining distinctly modern cat‐like features, including the reduction in size and lateral flattening of their teeth, putting them much more in line with modern conical teeth (Antón, 2013). The first metailurines, appearing approximately 11.6 Mya, also displayed a modern cat‐like postcranial morphology, with long limbs that likely made them adept at leaping (Antón, 2013). Found across North America, Eurasia, and Africa, the tribe persisted into the Pleistocene with the other machairodonts.

Smilodontini is the clade that epitomizes the saber‐tooth cat. Diverging from their common ancestor with metailurines approximately 10 Mya, the first smilodontines, including *Promegantereon ogygia* and *Paramachaerodus orientalis* were similar to the basal members of all the felids (Salesa et al., 2010, 2012) and resembled a modern leopard in build (Antón, 2013). The robustly built smilodontines appear at approximately 5 Mya, with *Megantereon* being found globally, whereas the famous *Smilodon* was restricted to the Americas (Kurten & Werdelin, 1990). It is the genus *Smilodon* that survived the latest into the Pleistocene, dying out approximately 11 Kya (Fiedel, 2009).

No extant feliforms are thought to possess true saber‐tooth morphologies, instead having acquired a different design of carnivoran canine teeth: conical, with a rounded cross‐section forming a backwards curving cone shape. Martin (1980) states that this morphology does not appear in the fossil record until the Early Miocene, approximately 20 Mya, but recently it has been proposed that some species in the group Nimravidae may have possessed this morphology in the Eocene to Oligocene, approximately 10 million years earlier (Barrett, 2021).

Although the broad morphologies of saber‐teeth have been described qualitatively, these descriptions have rarely been tested by quantitative data on tooth morphology. While linear measurements of the skull and canine morphology within the saber‐tooth feliforms have been used as phylogenetic characters in the past, a quantification of canine shape has not been attempted. Similarly, numerous studies focused on the functional morphology and physical properties of the cranial features of saber‐toothed species that allowed them to hunt and kill with their often impractical‐looking upper canine teeth (e.g. Christiansen & Wroe, 2007; Emerson & Radinsky, 1980; Figueirido et al., 2018; Janis et al., 2020; Piras et al., 2013). For example, work into functional morphology has been done to determine the stresses placed on the saber‐tooth skull when engaging in a variety of actions related to hunting and killing, determining that the teeth and skull of scimitar tooth species were more able to withstand the shaking motions of struggling prey, something dirk‐tooth species likely used their robust forelimbs to counteract (Figueirido et al., 2018). However, many of these studies fall into the same trap of small datasets with limited species variety, focusing on the abundant and well‐preserved specimens of *Smilodon fatalis* or *Homotherium latidens* as proxies for each saber‐tooth morphology, and therefore missing potentially novel results found in less‐studied, and less numerous, taxa. Lautenschlager et al. (2020) employed morphometric analysis to determine the variance in several of these functional characteristics, such as bite force, gape angle and bending strength of the mandible but did not include a quantification of the upper canine shape itself.

Here, we quantitatively analyzed the morphological differences in the upper canine teeth of the saber‐tooth feliforms using a two‐dimensional Geometric Morphometrics (GMM) approach and compared the data to the tooth morphology of extant feliforms. We further investigated the functional implications of upper canine shape using finite element analysis.

## 2 MATERIALS AND METHODS

### 2.1 Data collection

Tooth morphologies of the extinct species were sampled using images of the upper canine teeth of 27 saber‐tooth feliforms taken from existing literature (see Appendix S1). These included published reconstructions and images of specimens. Of the images and reconstructions consulted, only those which preserved a complete upper canine tooth, or featured one that could otherwise be easily reconstructed, were used. As far as could be ascertained only adult specimens with permanent (=non‐deciduous) upper canines were sampled. This resulted in specimens from all five major clades being represented. In order to standardize the images gathered for the subsequent Geometric Morphometrics analyses, the images of each tooth were sampled from the left lateral side or flipped if only the opposite side was available.

To compare the extinct data set, images of the upper canine teeth of 44 species of extant feliforms were taken from the literature, online databases, and primary data collection from the Lapworth Museum of Geology, Oxford University Museum of Natural History and the University of Cambridge Zoological Museum collections (see Appendix S1). These specimens span all seven families that make up the suborder of Feliformia, although the species sampled only make up 39% of all species within the suborder. This was intentional, as the focus of the study was on the extinct species, and therefore fully sampling all extant species was not necessary. Instead, a focus was put on sampling the family Felidae with the highest resolution (as three extinct clades are thought to fall within this family), and then the rest of the six families with less scrutiny. The primary data collection was done with the camera of a Huawei P30 Pro smartphone, which provided suitably high‐resolution images (10–20MP). These images were then flipped to assume the left‐facing position and cropped as necessary to focus on the tooth.

### 2.2 Geometric morphometric analysis

**FIGURE 1:**
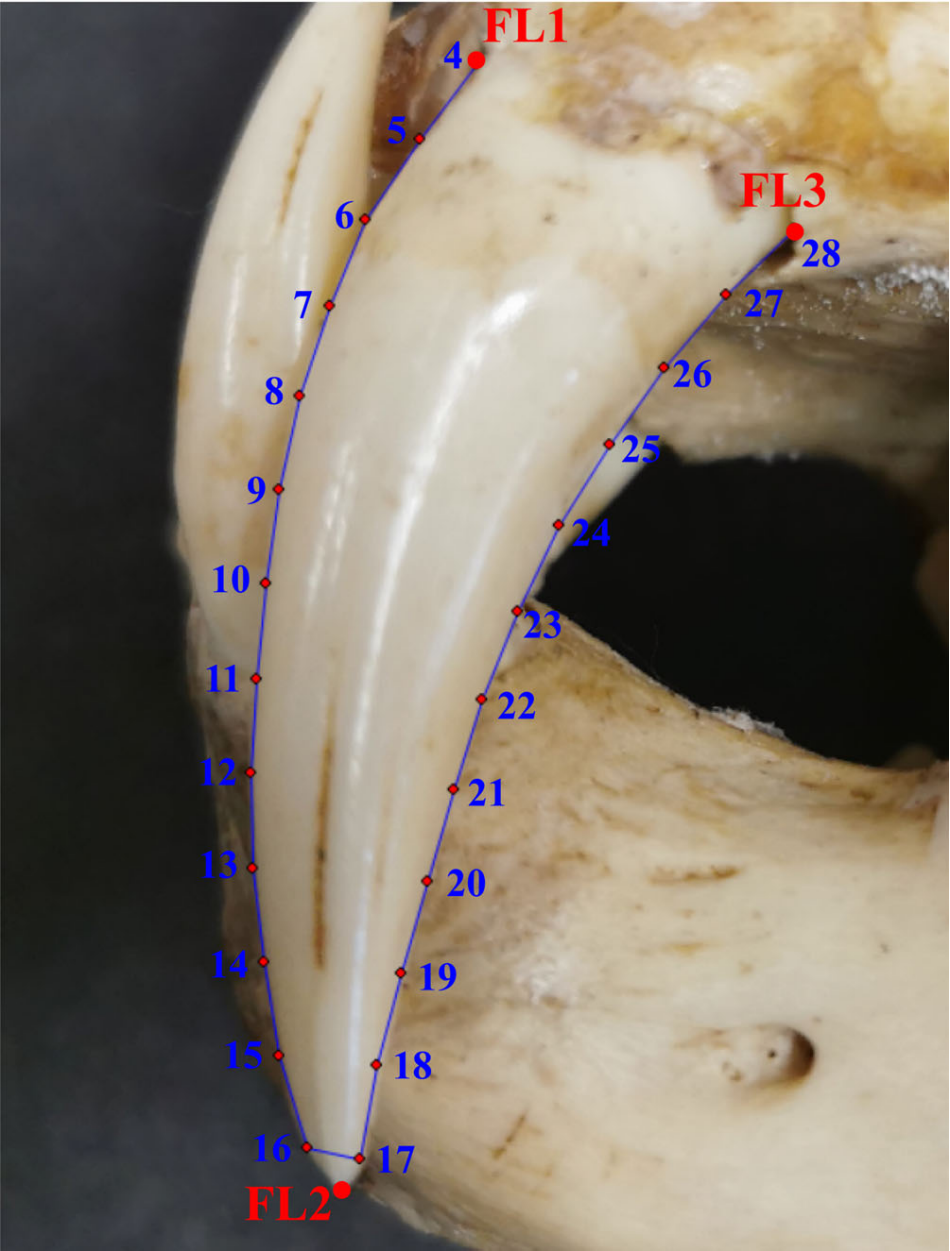
Position of the landmarks placed on each upper canine tooth, numbered sequentially. Fixed landmarks (FL) are marked in red, and semi‐landmarks (SL) are marked in blue. Exemplified by an image of *Neofelis nebulosa*.

For the shape quantification using a geometric morphometrics (GMM) approach three fixed landmarks were placed at the mesial contact between the upper canine and the maxilla, the tip of the upper canine, and the distal contact between the upper canine and the maxilla (Figure 1). In addition, 25 semi‐landmarks were placed on the mesial and distal edges between the fixed landmarks for each tooth. The start and end positions for the semilandmarks were placed in close proximity to fixed landmarks one and three as reference points. After placing all semilandmarks, the “resample curves by length” option inf tpsDig2 was used to place them at equidistant points. All landmark collection was completed in tpsDig2 (Rohlf, 2004). The full landmark data file for all species is available as Appendix S1. The landmark data (28 coordinates for each specimen) generated via GMM was subsequently superimposed using Procrustes analysis to standardize the data and then subjected to a principal component analysis (PCA) in PAST 4.05 (Hammer et al., 2001).

### 2.3 Finite element analysis

To assess the biomechanical performance of the selected upper canine tooth shapes, finite‐element analyses (FEA) were performed. Different upper canine tooth shapes evenly spread across the morphospace plots were extracted using tpsRelw 1.49 (Rohlf, 2010). For the extinct species, 12 upper canine models and for the combined data set, 20 upper canine models were created. Some of these represent theoretical shapes not occupied by sampled species but were included to assess their functional morphology.

All extracted shapes were modeled as extruded models (following Morales‐García et al., 2019) using a box‐modeling approach (Rahman & Lautenschlager, 2016) in Blender 3.5 (blender.org). The models were extruded in the third dimension by a consistent width of 1 mm. For FEA, the extruded models were exported from Blender as .STL files and imported into HyperMesh 11 (Altair) for solid meshing and the setting of boundary conditions. Mesh size was kept uniform to generate a quasi‐ideal mesh following Marcé Nogué et al. (2013). All models were assigned isotropic material properties (*E* = 38.6 GPa, *ʋ* = 0.4; Figueirido et al., 2018). Two functional scenarios were tested. (i) A puncture scenario with a single dorsally directed nodal force applied to the tip of the upper canine tooth. (ii) A second set of analyses were performed representing a pull‐back scenario with a force applied in mesial direction to the distal margin of the upper canines. For both scenarios, load forces were scaled following the quasi‐homothetic transformation approach Marcé Nogué et al. (2013), which ensures correct force/surface area scaling for extruded models as used here. Models were further constrained from movement at six nodes in *x*‐, *y*‐ and *z*‐directions at the base of the tooth models. All models were imported into Abaqus 6.141 (Simulia) for analysis and post‐processing.

## 3 RESULTS

### 3.1 GMM analysis—extinct dataset

**FIGURE 2:**
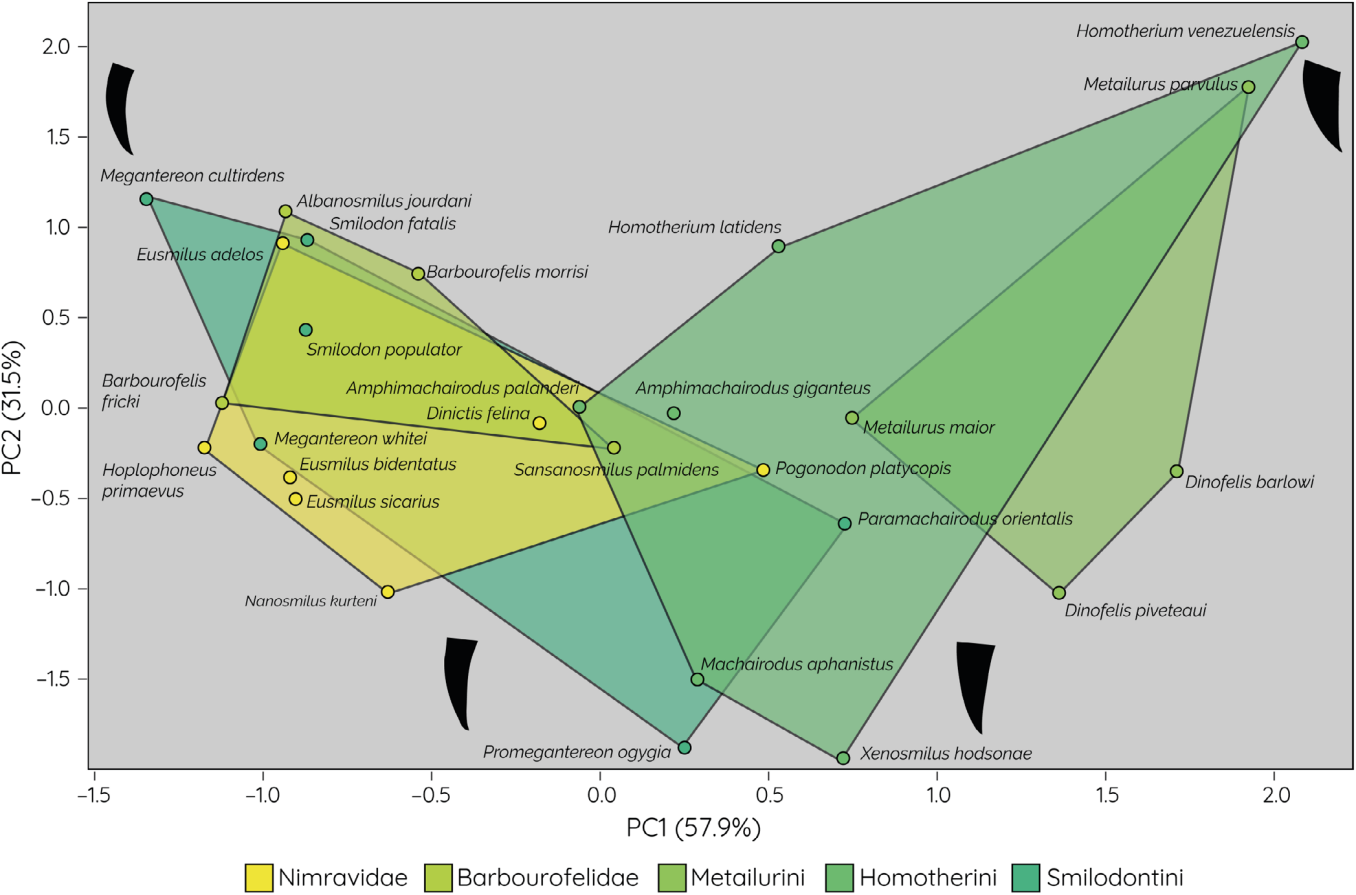
Morphospace plot showing the distribution and morphospace occupation of different fossil saber‐tooth species and clades. Extreme morphologies represented by silhouettes of the upper canines.

Upper canine tooth morphology varies significantly across the saber‐tooth feliforms. This variance is explained predominantly by PC1 (57.9%) and PC2 (31.5%), with these components combined explaining 89.4% of variance. PC1 is generally analogous to tooth length and width, with a negative value representing long and narrow teeth (Figure 2). PC2 can be interpreted to represent tooth curvature, with the more negative values representing a generally “straighter” tooth with less dramatic curving.

In terms of morphospace occupation by clade/species, the majority of barbourofelids fall within a relatively small area of the PCA plot (negative PC1, positive PC2), representative of large, highly recurved upper canines. *Sansosmilus palmidens* expands the barbourofelid morphospace negatively along PC2 and positively along PC1. Nimravidae largely overlap with barbourofelids, mostly due to *Eusmilus adelos* expanding the morphospace positively along PC2. All other nimravids occupy a position along negative PCs, mostly in the lower left of the PCA plot representing a long but less highly curved upper canine morphology. *Pogonodon platycopis* is the only nimravid species that scores positively on PC1. Smilodontini partially overlaps the morphospace of Barbourofelidae and Nimravidae but is separated into two main clusters: the long and highly curved *Smilodon* and *Megantereon* species scoring negatively along PC1, whereas *P. orientalis*, and *P. ogygia* expand the clade's morphospace towards positive PC1 and negative PC2. Homotherini occupies a large morphospace area but with only one species from Homotherini scoring negatively on PC1 (this being *Amphimachairodus palanderi*). This species also results in the hull of this clade to overlap with those of every other clade, although the overlap only contains a single species each from the other groups. Homotherini also contains the species with the most positive PC1 and PC2 values, both seen in *Homotherium venezuelensis*. This species diverges from its most morphologically similar clade mates on PC1 and on PC2, representing the shortest upper canines with the highest curvature in the sample. The species with the most negative PC2 value, and therefore upper canines with the least curvature, is *Xenosmilus hodsonae*. The Metailurines are the group with the most isolated position in the morphospace with their hull only overlapping with one other clade (Homotherini). They are the only clade where all members score positively on PC1. Through PC2 they are more diverse, with all but *Metailurus parvalus* scoring negatively on this axis. The two species of *Dinofelis* represent a unique morphology of upper canines with low curvature.

### 3.2 GMM analysis—combined dataset

**FIGURE 3:**
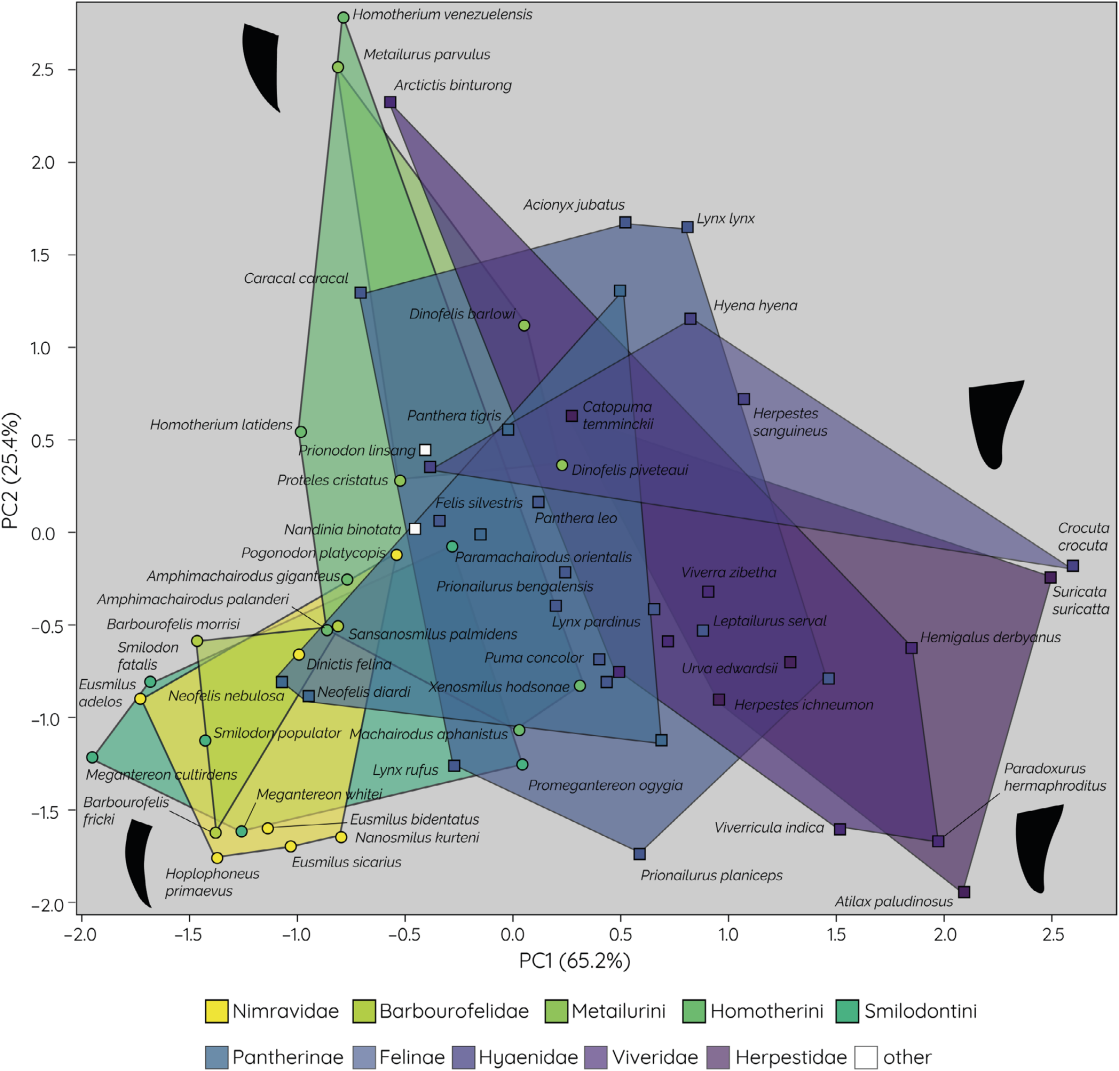
Morphospace plot showing the distribution and morphospace occupation of different fossil saber‐tooth species (dots) and extant felids (squares). Extreme morphologies represented by silhouettes of the upper canines. Species mentioned in the text are labeled with their scientific names.

The combined dataset including both the extinct and extant species shows a similar pattern for the fossil saber‐tooths but with the modern groups expanding the morphospace along PC1 (note, that the plot appears rotated counter‐clockwise compared to the PCA of the extinct species in Figure 2). Principal components 1 and 2 explain 90.6% of the variance within this dataset (PC1 = 65.2%, PC2 = 25.4%) (Figure 3). PC1 represents tooth curvature, with a more negative score representing a higher curvature, whereas PC2 represents the tooth aspect ratio in this dataset, with a negative value representing relatively elongate and narrow upper canines. From this, it can be concluded that upper canine curvature explains a greater amount of variance in this dataset than relative tooth length, and also explains a greater amount of variance in this combined dataset than in the dataset of extinct species.

The morphospace representing the very long and highly curved upper canines (scoring negatively on both PC1 and PC2) is occupied almost exclusively by extinct species, with the exception being the two species of the genus *Neofelis*, the clouded leopards. They fall closest to the nimravid *D. felina*, though notably fall more negatively than that species in both components. The only other extant species that scores negatively in both components is *Lynx rufus*, the bobcat, and it is the only other extant species (other than *Neofelis*) that overlaps with the hull of Smilodontini. In the positive PC2/negative PC1 morphospace (indicating a long upper canine with low curvature), several extant species fall within the hulls of extinct clades. Within the hull for Homotherini, seven extant feliform species represent three separate clades: Felinae, Pantherinae, and Hyaenidae. Due to its overlap with Homotherini, the hull for Metailurini also shares the previously mentioned clades, although the pantherine species represented in the hull is different, *Panthera tigris* (the tiger) being present in Metailurini compared to *Panthera leo* (the lion) in Homotherini.

Conversely, a great number of extinct species fall close to extant feliforms in other areas of morphospace. The most anomalous extinct species *H. venezuelensis* and *Metailurus parvulus* continue to occupy a unique area of morphospace, with the viverrid *Artictis binturong* (the binturong) falling the closest (though more positively on PC1 and more negatively on PC2) and *Caracal caracal* (the caracal) scoring similarly on PC1, but much more negatively on PC2. Only five extinct species (representing the clades Smilodontini, Homotherini, and Metailurini) fall positively on PC1. All of the species fall within the hull for Felinae. Two species, *Dinofelis piveteaui* and *X. hodsonae*, also fall within the hull for Pantherinae in positive PC1. The species closest to *X. hodsonae* is not a pantherine, however, instead being *Puma concolour*, the mountain lion (Felinae).

*Metailurus major* is unique in these results as it falls closer to species outside of the family Felidae (which it occupies) than any species within it. *Proteles cristata* (Aardwolf, family Hyaenidae) and *Prionodon linsang* (banded linsang, family Prionodontidae), and to a slightly lesser extent, *Nandinia biontata* (African Palm civet, family Nandiniidae) all fall closer to *M. major* than any other extant or extinct feliforms.

Observations can also be made between extant feliforms. Species from all extant clades other than Europlearidae (*Cryptoprocta ferox*, the fossa) are found within the hull for Felinae, and the family Felidae as a whole when the Pantherinae hull is included. This shows a fairly circular hull suggesting an “average” morphology for extant feliforms that generally falls slightly negatively on PC1 and positively on PC2. An observed similarity of note is the two species that score most positively on PC1, *Crocuta crocuta*, the spotted hyena, and *Suricata suricatta*, the meerkat. This highly positive PC1 score suggests a very short upper canine, and as they both score close to zero for PC2 they exhibit an “average” curvature. Another cross‐clade morphological similarity is seen between *Catopuma temminckii*, the Asian Golden Cat (Felinae) and *Herpestes saguineus*, the slender mongoose (Herpestidae).

### 3.3 Functional analyses

**FIGURE 4:**
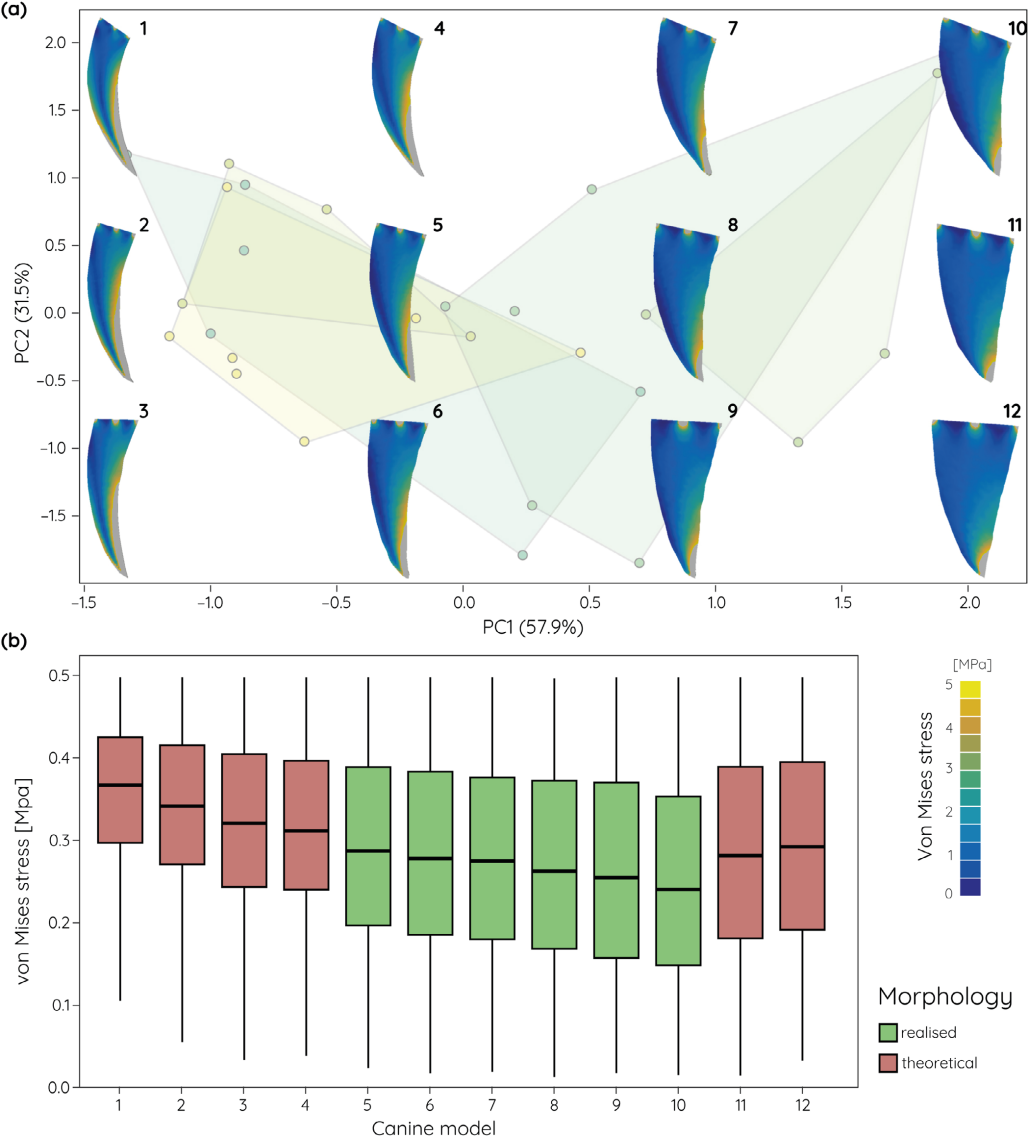
Finite element analyses results for an upper canine stabbing scenario of theoretical and actually realized upper canine models. (a) von Mises stress contour plots superimposed onto the morphospace (see Figure 2) for fossil saber‐tooth species. (b) Box plots for average von Mises stress values.

**FIGURE 5:**
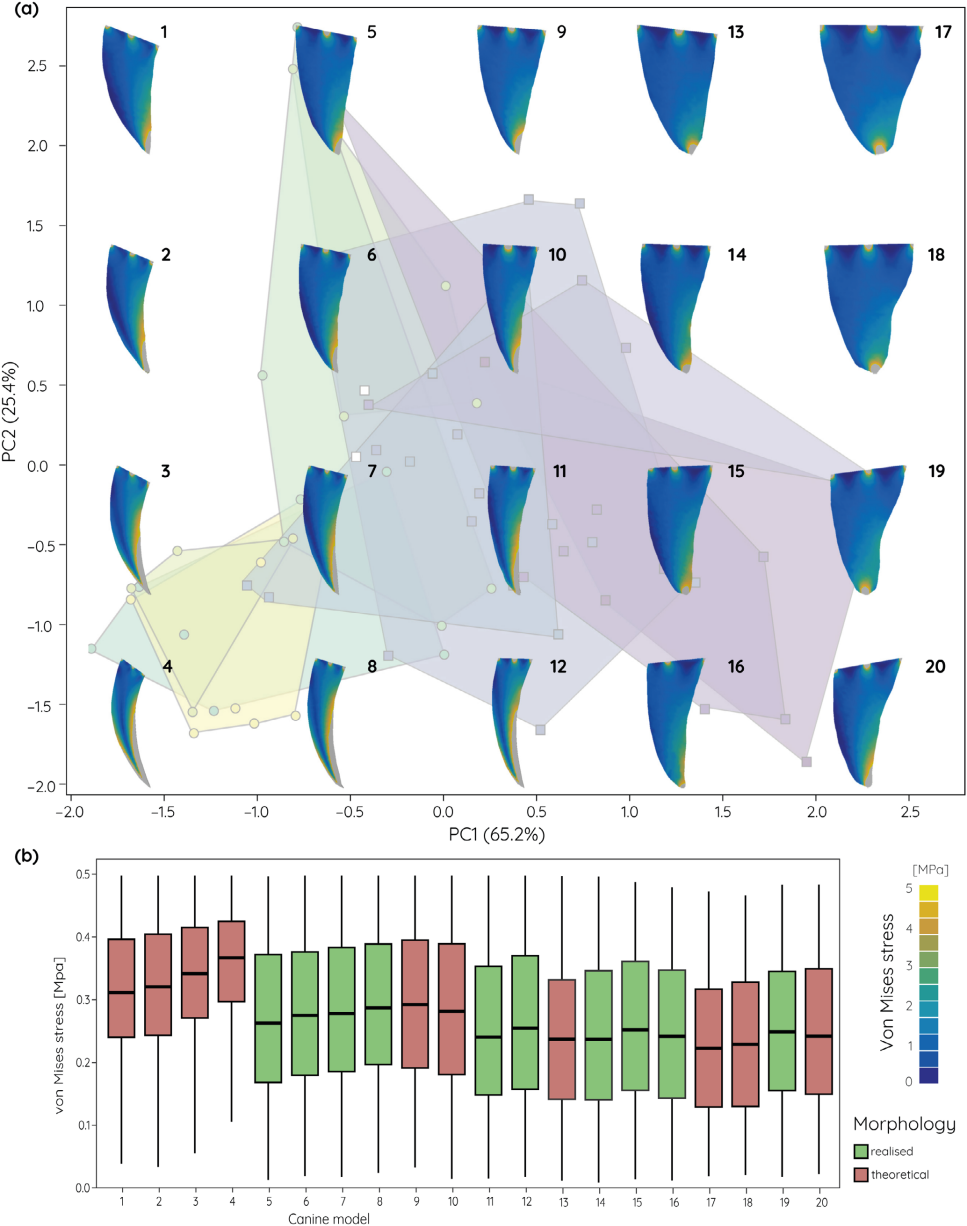
Finite element analyses results for an upper canine pulling scenario of theoretical and actually realized upper canine models. (a) von Mises stress contour plots superimposed onto the morphospace (see Figure 2) for fossil saber‐tooth species. (b) Box plots for average von Mises stress values.

The biomechanical analyses of the actual and theoretical upper canine models show that tooth shape represents a compromise between stress susceptibility and material investment. For the extinct data set (Figures 4 and 5), actually realized upper canine shapes are moderately curved to conical with correspondingly moderate to low stress values. In contrast, the theoretical upper canine shapes include mesiodistally narrow and strongly curved models along negative PC1. These models show comparatively high stress values for the stabbing (Figure 4) and pulling (Figure 5) scenarios, particularly along the distal margin of the upper canines. On the other end of the spectrum, theoretical shapes include broad and straight upper canines. These exhibit only very low stresses for both tested loading scenarios.

**FIGURE 6:**
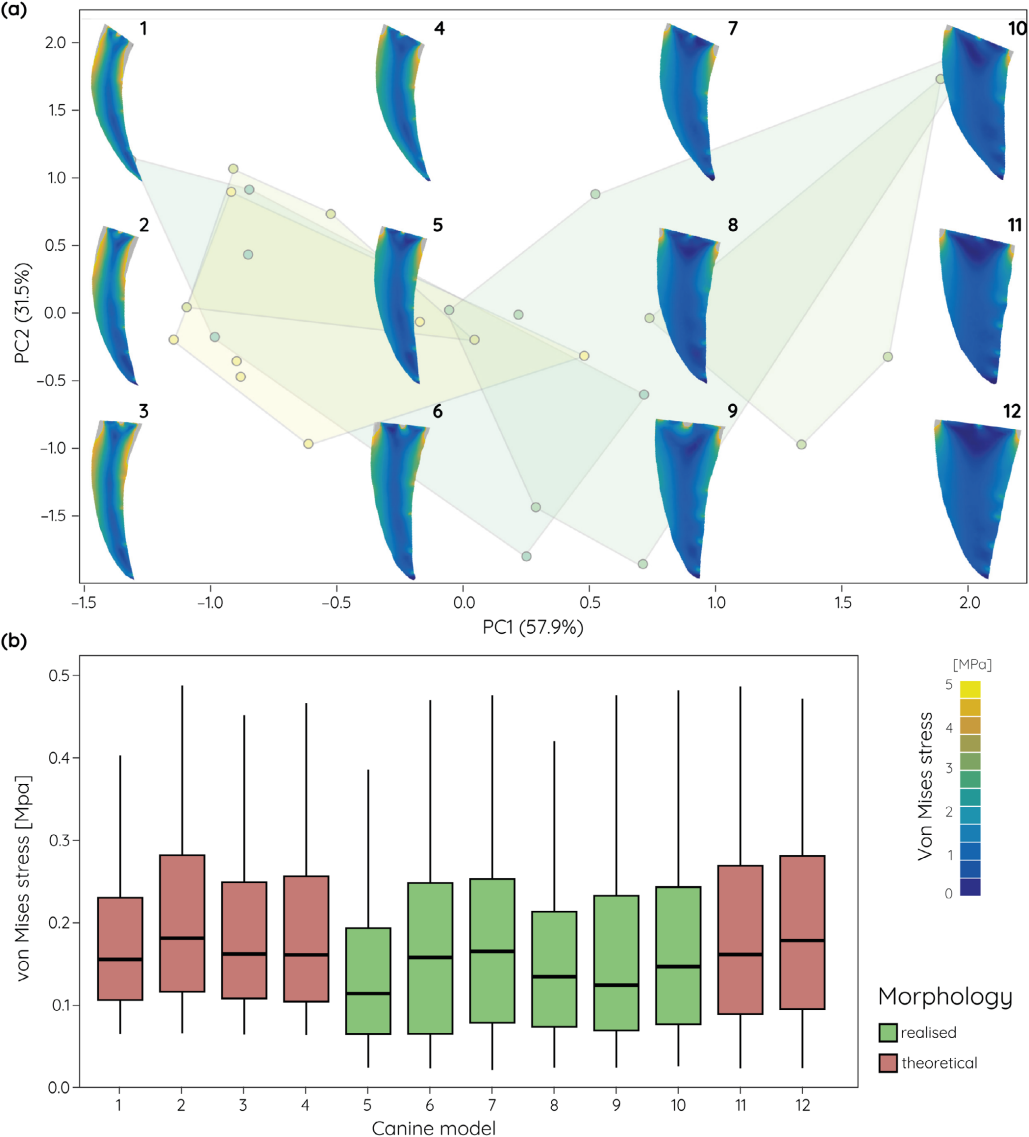
Finite element analyses results for an upper canine stabbing scenario of theoretical and actually realized upper canine models. (a) von Mises stress contour plots superimposed onto the morphospace (see Figure 3) for fossil and extant species. (b) Box plots for average von Mises stress values.

**FIGURE 7:**
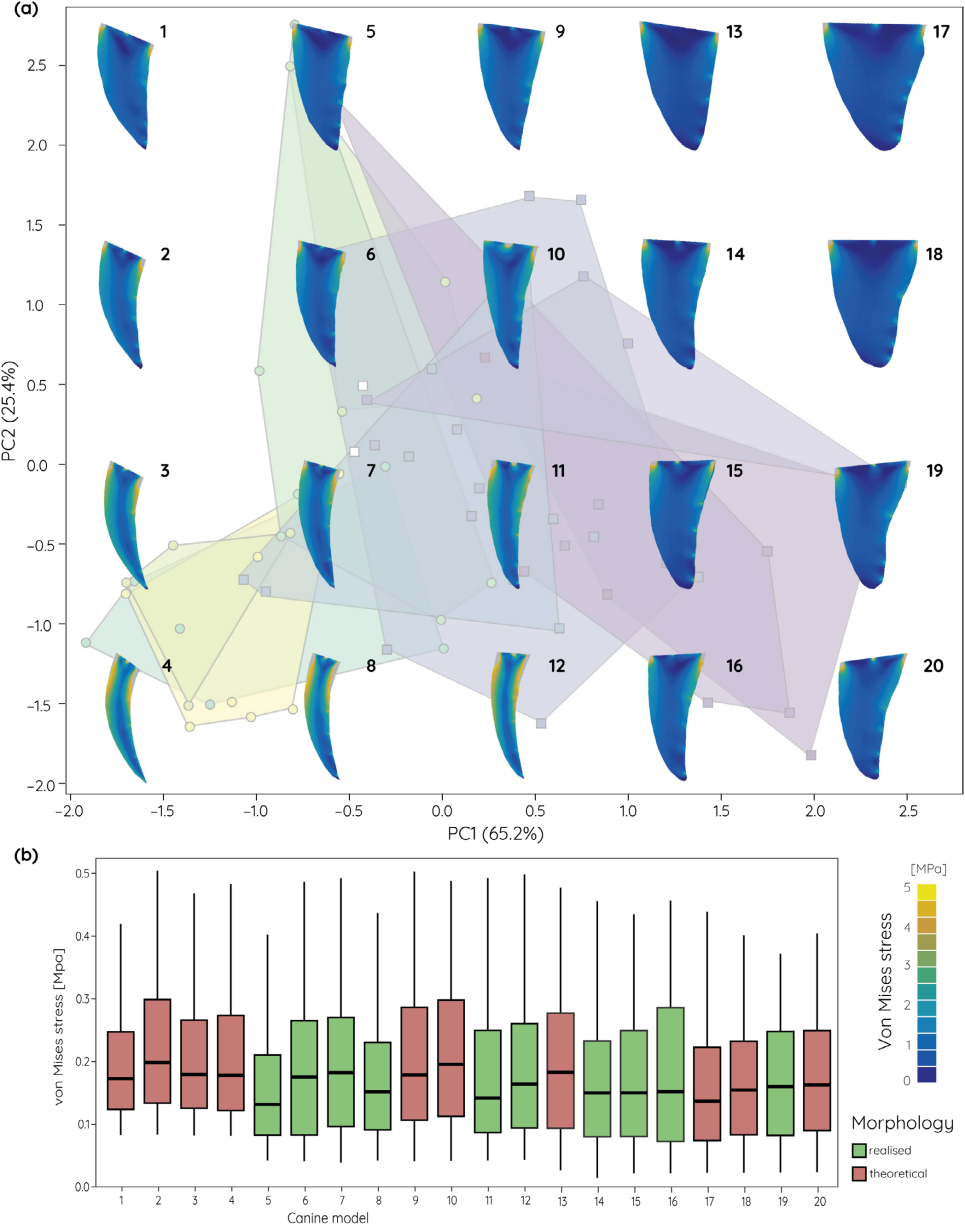
Finite element analyses results for an upper canine pulling scenario of theoretical and actually realized upper canine models. (a) von Mises stress contour plots superimposed onto the morphospace (see Figure 3) for fossil and extant species. (b) Box plots for average von Mises stress values.

The same pattern can be observed for the data set combining extant and extinct species (Figures 6 and 7). In particular along PC2, with very broad and only moderately curved or straight models occupying the theoretical morphospace, stress values are considerably lower.

## 4 DISCUSSION

It can be seen throughout extinct and extant feliforms that upper canine tooth morphology is considerably varied, and when analyzed in two dimensions, can be described using two key metrics: tooth length, and tooth curvature. This presents several avenues of discussion for both extinct and extant taxa, though focusing on different areas. In the extinct taxa, this analysis allows for the differences in upper canine morphology in the basal and derived taxa of different clades to be observed and commented on, and the similarities of the results of this study with ideas already presented in the literature to be identified. The combined dataset in contrast offers insights on the validity of upper canine shape as a defining characteristic, specifically for the saber‐tooths of Homotherini and Metailurini, and the possible wider functional implications of morphological similarity between extant and extinct species.

### 4.1 Upper canine morphology

Despite falling under a broad moniker, the morphology of saber‐tooth upper canines is very diverse. As observed in Lautenschlager et al. (2020)'s investigation into saber‐tooth functional morphology, there is no clearly observable close grouping of species into two morphologies, as would be suggested by the categories of “dirk teeth” and “scimitar teeth” (Kurtén, 1968). In this study, species with dirk‐tooth morphologies do fall within an area of morphospace occupied by the more derived smilodontines and barbourofelids, but the species are still not closely grouped together, and these clades also have members which fall far from this space. In fact, no clade has all of its members occupy a distinct and separate morphospace region, further exemplifying the level of morphological variability seen in saber‐teeth, even within clades thought to possess a single tooth type. Homotherini is a clear example of this, having the most diverse tooth morphology of clades sampled, spanning the largest amount of morphospace along both PC axes.

The results obtained in this study further support the work of Barrett (2021) who suggested that the nimravids did not follow a single evolutionary path to acquire saber‐teeth, instead diversifying into a range of morphologies and body sizes, likely to allow for niche partitioning within their environments. There is a distinct morphological partition between the members of Nimravinae (*D. felina* and *P. platycopis*) with shorter scimitar‐like upper canine and the members of Hoplophoninae (*Eusmilus* and *Nanosmilus kurteni*) acquiring longer dirk‐like upper canine, observed within the phylogenetic analysis in Barrett (2021), and in the morphospace plots here. Both of these groups lived contemporaneously, and their morphological divergence mirrors that of the true cats of Machairodontidae, though with many members of the clade being far smaller than the Pleistocene felids (Barrett, 2021). However, as the oldest clade analyzed in this study, sampling nimravid upper canine in sufficient preservation was difficult, and therefore the dataset is likely not representative of the true diversity the clade is known to exhibit.

In this context, the homotherine *X. hodsonae* is particularly interesting, as it has been described previously as a “scimitar toothed cat with dirk‐tooth features”, specifically in its postcranial skeleton, as it likely displayed the robust, almost bear‐like appearance of the derived dirk‐tooth smilodontines (Martin et al., 2000). This assessment is supported here with *X. hodsonae* falling close to the most basal smilodontines and in the opposite area of morphospace to other dirk‐toothed species. However, it also does not share a morphospace with other derived homotherines, being most similar to the most basal homotherine species *Machairodus aphanistus* (though even this specimen exhibits an increased upper canine length).

Although it is generally assumed that all of Smilodontini exhibit the dirk‐tooth morphology, there is in fact a significant shift in tooth morphology over their evolution, with the more basal taxa *P. ogygia* and *P. orientalis* displaying significantly shorter upper canines with low curvature. This morphology is also seen in the most basal members of the other saber‐tooth felid tribes, suggesting that the primitive condition for the last common felid ancestor was medium length upper canines with low curvature. The derived traits for the three tribes, however, are entirely different. For the smilodontines, the evolutionary destination is the long and highly curved dirk‐like upper canines, whereas for the metailurines, the derived morphology is a very short upper canine with low curvature, closely akin to modern feliforms. This study shows that through the evolution of the tribe, the derived genus *Dinofelis* abandons traditional saber‐tooth upper canine morphologies. *D. piveteaui* is regarded as the more traditionally “saber‐tooth‐like” of the two species, with the characteristic shortening of the hindlimbs seen in the other members of Machairodontidae (Antón, 2013). This is somewhat apparent in our analysis, as it falls closer to the homotherines in morphospace, but it is obviously still markedly distinct from their morphological destination. The homotherines first appear much more cryptic in their trajectory, but when *X. hodsonae* is excluded, a similar trend of derivation can be seen. When viewed in evolutionary order, the clade displays a movement positively in both principal components, ending in the acquisition of short but highly recurved upper canines unlike anything it would have existed contemporaneously with. *M. parvulus* died out approximately 3 million years before *H. venezuelensis* is thought to have lived (Piras et al., 2013), showing another re‐acquisition of a previous morphology.

Morphological overlap is also seen with extinct species into extant morphospaces, most prominently in *D. piveteaui* and *X. hodsonae* which both fall within the hull for Pantherinae. This is particularly unexpected, as these two species are considered the members of their clades that represent the most derived saber‐tooth traits (such as a shortening in the hind legs and lateral flattening of the upper canines) and therefore post‐cranially moving away from a pantherine form (Antón, 2013). *X. hodsonae's* upper canine morphology is most similar to that of the felid *P. concolour*, a species that shows opposite non‐dental features to the homotherine, such as a small, rounded skull and shorter forelimbs (Turner & Antón, 1997). The concentration of other members of Felinae around this morphospace also suggests that this upper canine shape has been acquired multiple times and is successful for a variety of diets, as the omnivorous viverrid *Genetta maculata* (Angelici, 2000) occupies a similar morphospace as the obligate carnivore felids.

The morphological similarity of *M. major* and *P. cristata* (the aardwolf) raises an interesting point when considering the extent to which feeding behavior can be predicted or assumed by tooth morphology. Like all felids, *M. major* was almost certainly an obligate carnivore, but the aardwolf is entirely insectivorous, and other than the upper canine teeth (thought to be retained for fighting), has dramatically reduced dentition in response to this diet (Koehler & Richardson, 1990). This extreme difference in diet is not apparent through this study's analysis, but this type of anomalous diet whilst retaining expected dental features is not uncommon, famously also seen in the Giant Panda (Figueirido et al., 2011). Therefore, palaeontological studies should keep this kind of variation in mind when attempting to determine the diets of extinct taxa, in particular when the research is only based on isolated dental material.

Among the extant taxa, a noteworthy result from this analysis is the position of the genus *Neofelis* in the morphospace of the extinct saber‐tooths. It has already been found that *Neofelis nebulosa* has longer upper canine teeth relative to skull length than any other extant felid, as well as displaying a similar level of lower canine reduction and other cranial characters seen in the extinct saber‐tooths (Christiansen, 2006), meaning its position in morphospace is not unexpected, but it is interesting that the genus' PC2 values related to curvature, also confirm their morphological similarity to extinct saber‐tooths, in particular, the closeness of this genus to *D. felina*. The possible similarity between these species has been remarked on previously in relation to their canine proportions however, the genus does not exhibit the lateral flattening of the canine teeth as seen in extinct saber‐tooths (Christiansen, 2006). Nevertheless, this added facet of morphological similarity to the extinct saber‐tooths continues to mark *Neofelis* as an important genus for use in comparative studies.

### 4.2 Upper canine function

The functional analyses of the simplified upper canine tooth models show that more elongate, narrow, and strongly recurved morphologies are more susceptible to increased stress, whereas shorter and broader teeth are less prone to stresses. This is not unexpected but confirms results from studies using three‐dimensional models of the whole cranium (Figueirido et al., 2018), but also of isolated canine teeth of non‐saber‐tooth carnivorans (Pollock et al., 2022). In particular, for simulations of stabbing behavior the stress values show distinct differences between the different extremes in upper canine shape. Interestingly, the theoretical models tested here not only show and exaggerated morphology but also increases or decreases in stress susceptibility; upper canines that exceed the curvature and length observed in fossil saber‐tooth species, whereas extremely broadened and straight upper canines show reduced stress values. This suggests that fossil saber‐tooth upper canines may have reached biomechanically and physiologically constrained limits. While strong curvature would increase the edge length compared to overall tooth length thereby providing an increased cutting surface, the increased stress susceptibility would render the upper canines more unstable and prone to breakage. On the other hand, a broad and straight upper canine morphology would provide increased tooth strength but would also require additional physiological investment in tooth material. Upper canine shape in the saber‐tooth carnivorans therefore represents a compromise between killing‐effectiveness, stress‐reduction, and material investment based on preferred prey choice. These results correspond well with findings from Pollock et al. (2021) on upper canine sharpness and robustness. In non‐felid carnivorans, tooth robustness increases with the hardness of food materials.

In this context, it should be noted that upper canine sharpness will facilitate tooth penetration into the prey. However, as tooth penetration is not reflected in our biomechanical analyses, the upper canine models could represent higher stress than would actually occur during upper canine stabbing. Tooth sharpness could provide a mechanism for saber‐tooth species with elongate and strongly recurved upper canines to avoid increased stress in such a scenario; sharp canines have been shown to be present in felids and species with a meat‐rich diet (Pollock et al., 2021). However, tooth sharpness would provide no functionally beneficial effects for pulling or lateral shaking scenarios using upper canine teeth which have been assumed unlikely for saber‐tooth species, such as *S. fatalis* (Brown, 2014; McHenry et al., 2007; Valkenburgh et al., 1990). Rather, these species would have relied on forelimb strength for prey fixation (Meachen‐Samuels & Van Valkenburgh, 2010) and harnessed the incisors and mandible during killing and feeding (Biknevicius et al., 1996; Chatar et al., 2022).

The results of previous bite force analyses of both extinct and extant taxa offer further comparative data to the results of this study. It can be observed in the extinct species that a more positive PC1 is linked to a higher relative bite force, as calculated by Lautenschlager et al. (2020). Species such as the derived smilodontines and barbourofelids that scored very negatively in PC1 exhibited low relative bite forces in their study, likely thought to be driven by a shift towards a canine‐shear bite killing method, which utilizes the neck musculature rather than a high bite force (Brown, 2014; McHenry et al., 2007). Conversely, the increase in PC1 in metailurines mirrors an increase in relative bite force in the clade over time, suggesting a movement towards a novel killing strategy, or a specialization for small prey.

Among extant species, *C. crocuta* marks the most positive PC1 of species sampled, likely due to its bone‐crushing feeding behavior selecting for shorter more robust upper canines, and the specimen sampled having worn its tooth down in this manner when alive. However, Christiansen and Wroe (2007) calculated that *C. crocuta* does not have the highest bite force of extant feliforms, instead having a value that is considered average for their body size and lower than that of species that fall more negatively on PC1, such as the pantherine cats. This is to say that an increase in PC1 does not necessarily directly correlate to an increase in bite force, but instead that a need for high bite force for behaviors such as durophagy is related to short, robust upper canines that fall high on PC1.

### 4.3 Limitations

There are several limitations in this study that should be acknowledged. Firstly, a defining characteristic of saber‐teeth has been overlooked in the 2D method of data collection, this being lateral flattening. The flattened cross‐section of the upper canines is a key feature that separates them from the conical upper canines of the modern feliforms (Martin, 1980), as well as distinguishing the two morphologies of saber‐teeth from one another. Therefore, not having the ability to include this “cross‐sectional width” measurement or adapt the geometric morphometric analysis to work with 3D landmarks affects the usefulness of this data to a significant degree by removing a key diagnostic feature from each specimen. When discussing the variation of upper canine morphology between the smilodontines and homotherines, for example, Antón (2013) specifically mentions the flattening of the homotherine upper canines as a derived trait distinct to the tribe. In addition to this, it has also been found that morphological similarity alone can be a poor indicator of actual function, especially when used across clades that display convergent traits (Lautenschlager et al., 2016).

## 5 CONCLUSIONS

This study provides a new morphometric dataset to the field of saber‐tooth feliform research, and in turn is able to support previous research into the morphological variation and evolution of these enigmatic species. The saber‐tooth ecomorph of dirk teeth can be seen prominently in the morphospace plot, but there does not appear to be a distinct scimitar‐tooth morphology held by any species. Instead, basal taxa in each clade gather in a neutral morphospace, and then radiate out into unique morphologies for each genus. In addition, a similarly novel dataset is generated for the extant feliforms, highlighting the morphological diversity within this group, and how the upper canine morphologies of the extinct saber‐tooths diverge and converge with those alive today. Significant overlap between the morphologies of extinct and extant taxa can be observed, specifically the morphology of *N. nebulosa* falling within the hulls of three dirk‐toothed clades, and the overlap of the homotherines and metailurines with modern felids and pantherines. The similarity of upper canine tooth shape across varying dietary niches can also be seen, with omnivorous and insectivorous species occupying similar morphospace to extant and extinct obligate carnivores, therefore highlighting the potential for extinct diets to be more diverse than upper canine tooth morphology may first imply. Saber‐teeth upper canines further represent a functional compromise between sharpness, curvature, and length on the one hand, and robustness and material investment on the other.

## AUTHOR CONTRIBUTIONS

**Stephan Lautenschlager:** Conceptualization; data curation; supervision; investigation; project administration; writing – review and editing; resources; validation; methodology; formal analysis; visualization. **Caitlin D. Shelbourne:** Formal analysis; visualization; writing – original draft; methodology; investigation; writing – review and editing.

## CONFLICT OF INTEREST STATEMENT

The authors declare no conflicts of interest.

## Supporting information


**Appendix S1:** Species list.

## Data Availability

The data (FEA result files) that support the findings of this study are available via the following Figshare link: 10.6084/m9.figshare.25627491
